# Supplementary material for: Operationally classical simulation of quantum states
Source: Nat Commun. 2026 Jan 27;17:1104. doi: 10.1038/s41467-026-68581-3 (PMC12852923; doi:10.1038/s41467-026-68581-3)
Supplement: Supplementary file 1 — Supplementary Information [file 41467_2026_68581_MOESM1_ESM.pdf]

Supplementary Material: Operationally classical simulation of quantum states

Gabriele Cobucci,[1,†] Alexander Bernal,[2,†] Martin J. Renner,[3,4,5] and Armin Tavakoli[1,*]

[1]*Physics Department and NanoLund, Lund University, Box 118, 22100 Lund, Sweden.*
[2]*Instituto de Física Teórica, IFT-UAM/CSIC, Universidad Autónoma de Madrid, Cantoblanco, 28049 Madrid, Spain.*
[3]*ICFO - Institut de Ciencies Fotoniques, The Barcelona Institute of Science and Technology, 08860 Castelldefels, Barcelona, Spain*
[4]*University of Vienna, Faculty of Physics and VDSP, Vienna Center for Quantum Science and Technology (VCQ), Boltzmanngasse 5, 1090 Vienna, Austria.*
[5]*Institute for Quantum Optics and Quantum Information (IQOQI), Austrian Academy of Sciences, Boltzmanngasse 3, 1090 Vienna, Austria*
(Dated: January 5, 2026)

I. SUPPLEMENTARY NOTE 1: CONVEXITY OF THE SET $\mathcal{S}$

We show that the set $\mathcal{S}$ of all classically simulable sets of states is convex. To this end, let us consider two sets $\mathcal{E} = \{\rho_x\}_x \in \mathcal{S}$ and $\mathcal{E}' = \{\sigma_y\}_y \in \mathcal{S}$ and let us define $\mathcal{E}_p = \{p\,\rho_x + (1-p)\sigma_y\}_{x,y}$. We now prove that $\mathcal{E}_p$ admits a classical simulation for any $p \in [0,1]$.

Since $\mathcal{E} \in \mathcal{S}$, following Definition 1 of the main text we have

$$\rho_x = \int d\lambda\, q(\lambda)\, \tau_{x,\lambda}, \quad \forall x, \tag{1}$$

where $[\tau_{x,\lambda}, \tau_{x',\lambda}] = 0$. Similarly, for $\mathcal{E}' \in \mathcal{S}$

$$\sigma_y = \int d\mu\, \tilde{q}(\mu)\, \chi_{y,\mu}, \quad \forall y, \tag{2}$$

with $[\chi_{y,\mu}, \chi_{y',\mu}] = 0$. Call $\Gamma_\lambda := \{\lambda\}$ and $\Gamma_\mu := \{\mu\}$ the sets of all possible values of λ and μ in (1) and (2), respectively.

In order to find a classical simulation for $\mathcal{E}_p$, we define the set $\Gamma_\nu := \{\nu\} = \Gamma_\lambda \cup \Gamma_\mu$ and the associated probability distribution $Q(\nu)$:

$$Q(\nu) = \begin{cases} p\, q(\lambda) & \text{for} \quad \nu \in \Gamma_\lambda, \\ (1-p)\tilde{q}(\mu) & \text{for} \quad \nu \in \Gamma_\mu. \end{cases} \tag{3}$$

Similarly, let us define the states

$$\omega_{(x,y),\nu} = \begin{cases} \tau_{x,\lambda} & \text{for} \quad \nu \in \Gamma_\lambda, \\ \chi_{y,\mu} & \text{for} \quad \nu \in \Gamma_\mu. \end{cases} \tag{4}$$

Therefore, a classical simulation model for all the states in $\mathcal{E}_p$ is given by

$$\int d\nu\, Q(\nu)\, \omega_{(x,y),\nu} = p \int d\lambda\, q(\lambda)\, \tau_{x,\lambda} + (1-p) \int d\mu\, \tilde{q}(\mu)\, \chi_{y,\mu} = p\, \rho_x + (1-p)\, \sigma_y, \quad \forall (x,y), \tag{5}$$

with $[\omega_{(x,y),\nu}, \omega_{(x',y'),\nu}] = 0$. This implies $\mathcal{E}_p \in \mathcal{S}$.

II. SUPPLEMENTARY NOTE 2: CLASSICAL MODELS FOR PURE QUANTUM STATES WITH ISOTROPIC NOISE

A. Proof of Result 1

We give an explicit classical model of complexity r for simulating any set $\mathcal{E}$ of arbitrary cardinality, m, comprised of pure states, $\{\,|\psi_x\rangle\}_{x=1}^m \subset \mathbb{C}^d$, subject to isotropic noise,

$$\rho_x = v\,|\psi_x\rangle\!\langle\psi_x| + \frac{1-v}{d}\mathbb{1}_d, \tag{6}$$

[†] These authors contributed equally.

[*] armin.tavakoli@fysik.lu.se

for some visibility $v \in [0,1]$. The protocol to build the simulation is as follows:

1. Choose $\{\,|1\rangle\,,\,|2\rangle\,,\ldots,\,|r\rangle\}$ as an orthonormal basis of $\mathbb{C}^r$.
2. For a given $d \times d$ unitary transformation U, consider the new basis $\{U\,|1\rangle\,, U\,|2\rangle\,,\ldots, U\,|r\rangle\}$. Select the basis element that has the largest overlap with the state $|\psi_x\rangle$.
3. To simulate each ρ_x, we average over the Haar measure the as-above selected basis element.

Let us denote the element of the rotated basis that overlaps the most with $|\psi_x\rangle$ by $|i_U^{(x)}\rangle = U\,|i^{(x)}\rangle$. Thus, we are claiming that

$$\rho_x \stackrel{!}{=} \int d\mu_{\text{Haar}}\,(U)\;|i_U^{(x)}\rangle\!\langle i_U^{(x)}|\,. \tag{7}$$

The integral on the right-hand side is invariant under any unitary transformation U_x that leaves invariant the state $|\psi_x\rangle$. Namely, by definition of $|i_U^{(x)}\rangle$ and invariance of $|\psi_x\rangle$ under U_x:

$$\langle \psi_x | i^{(x)}_{U_x U}\rangle = \max_i\;\langle \psi_x | U_x U | i\rangle = \max_i\;\langle \psi_x | U | i\rangle = \langle \psi_x | i_U^{(x)}\rangle\,. \tag{8}$$

Hence, the state $|i^{(x)}\rangle$ remains the same after the unitary transformation:

$$\begin{aligned} U_x \rho_x U_x^\dagger &= U_x \left(\int d\mu_{\text{Haar}}\,(U)\,U\,|i^{(x)}\rangle\!\langle i^{(x)}|\,U^\dagger\right) U_x^\dagger \\ &= \int d\mu_{\text{Haar}}\,(U)\,U_x U\,|i^{(x)}\rangle\!\langle i^{(x)}|\,U^\dagger U_x^\dagger, \end{aligned} \tag{9}$$

and by the left and right invariance of the Haar measure:

$$\begin{aligned} U_x \rho_x U_x^\dagger &= \int d\mu_{\text{Haar}}\,(U)\,U_x U\,|i^{(x)}\rangle\!\langle i^{(x)}|\,U^\dagger U_x^\dagger \\ &= \int d\mu_{\text{Haar}}\,(U)\,U\,|i^{(x)}\rangle\!\langle i^{(x)}|\,U^\dagger = \rho_x. \end{aligned} \tag{10}$$

Since the only states invariant under these kinds of transformations are of the form

$$v\,|\psi_x\rangle\!\langle\psi_x| + \frac{1-v}{d}\mathbb{1}_d \tag{11}$$

for some visibility $v \in [0,1]$, we must have that the simulation actually gives a state of this specific form. In order to compute the associated visibility, we take the expectation value over $|\psi_x\rangle$:

$$\frac{(d-1)v+1}{d} = \int d\mu_{\text{Haar}}\,(U)\,\Big|\,\langle\psi_x|i_U^{(x)}\rangle\Big|^2. \tag{12}$$

Expanding the right-hand side we obtain the following sum:

$$\int d\mu_{\text{Haar}}\,(U)\,\Big|\,\langle\psi_x|i_U^{(x)}\rangle\Big|^2 = \sum_{i=1}^{r}\int_i d\mu_{\text{Haar}}\,(U)\,|\,\langle\psi_x|U|i\rangle|^2, \tag{13}$$

where the subscript i indicates that the integration is only over unitaries U such that $|\,\langle\psi_x|U|i\rangle|^2$ is greater than $|\,\langle\psi_x|U|j\rangle|^2$ for any other $j \neq i$. For example, if we consider $i = 1$ integral $\int_1 d\mu_{\text{Haar}}\,(U)\,|\,\langle\psi_x|U|1\rangle|^2$ is performed over the unitaries U for which $|\,\langle\psi_x|U|1\rangle|^2 \geq |\,\langle\psi_x|U|j\rangle|^2\;\forall j = 2,\ldots,r$. In addition, due to the left and right invariance of Haar measure, each integral in the sum gives the same result. One can see this by considering the unitary transformation that permutes elements in the computational basis. Hence, we can restrict to only compute the first one:

$$\int d\mu_{\text{Haar}}\,(U)\,\Big|\,\langle\psi_x|i_U^{(x)}\rangle\Big|^2 = \sum_{i=1}^{r}\int_i d\mu_{\text{Haar}}\,(U)\,|\,\langle\psi_x|U|i\rangle|^2 = r\int_1 d\mu_{\text{Haar}}\,(\psi)\,|\,\langle\psi|1\rangle|^2. \tag{14}$$

where in the last step we have further identified the integration over unitary transformations with that over pure states.

This last integral can be computed applying the techniques developed in [1, 2]. For instance, given an orthonormal basis $\{ |\phi_j\rangle\}_{j=1}^d$ we parametrize any non-normalized pure state $|\tilde{\psi}\rangle$ by

$$|\tilde{\psi}\rangle = \frac{1}{\sqrt{d}} \sum_{j=1}^{d} z_j |\phi_j\rangle , \tag{15}$$

where z_j are zero-mean Gaussian random variables with the properties $\langle z_j^* z_k\rangle = \delta_{j,k}$ and $\langle z_j z_k\rangle = 0$. Writing $|\tilde{\psi}\rangle = m|\psi\rangle$, we denote the measure over this set as $d\mu_G(\psi, m)$. It can be seen [2] that the measure factorizes as

$$d\mu_G(\psi, m) = d\mu_{\text{Haar}}(\psi)\, d\mu_G(m), \qquad \int d\mu_G(m)\, m^2 = 1. \tag{16}$$

Thus,

$$\int_1 d\mu_{\text{Haar}}(\psi) \,|\langle\psi|1\rangle|^2 = \int_1 d\mu_G(\psi, m) \left|\langle\tilde{\psi}|1\rangle\right|^2. \tag{17}$$

For simplicity, we define $z_j = \sqrt{u_j} e^{i\theta_j}$ so that

$$d\mu_G(\psi, m) = \frac{1}{(2\pi)^d} \exp\left\{-\sum_{j=1}^{d} u_j\right\} du_1 \cdots du_d d\theta_1 \cdots d\theta_d. \tag{18}$$

In addition, since $\{ |\phi_j\rangle\}_{j=1}^d$ is arbitrary we can take $|\phi_1\rangle = |1\rangle$. Hence, $\left|\langle\tilde{\psi}|1\rangle\right|^2 = \frac{u_1}{d}$ and the integration over the phases is trivial (see Appendix B.3 of [2] for more details). Concerning the integration over the modulus, we have to integrate u_1 from 0 to ∞, the next $r-1$ from 0 to u_1 (hence satisfying the constraint imposed by the domain of integration) and all the others again from 0 to ∞:

$$\begin{aligned} \int_1 d\mu_G(\psi, m) \left|\langle\tilde{\psi}|1\rangle\right|^2 &= \frac{1}{d}\int_0^\infty du_1 u_1 \int_0^{u_1} du_2 \cdots \int_0^{u_1} du_r \int_0^\infty du_{r+1} \cdots \int_0^\infty du_d \exp\left\{-\sum_{j=1}^{d} u_j\right\} \\ &= \frac{1}{d}\int_0^\infty du_1 u_1 e^{-u_1}\left(1 - e^{-u_1}\right)^{r-1} = \frac{1}{d}\frac{H_r}{r}. \end{aligned} \tag{19}$$

with $H_r = \sum_{k=1}^r 1/k$ the harmonic number. To evaluate the last integral we have used the result derived in Appendix B.3 of [2]. Finally,

$$\frac{(d-1)v + 1}{d} = r\frac{1}{d}\frac{H_r}{r} = \frac{H_r}{d} \implies v = \frac{H_r - 1}{d-1}. \tag{20}$$

B. Improved simulation for states in lower-dimensional subspaces

The previous simulation is valid for any noisy set. However, it can be further improved when the initial m pure states span an s-dimensional space with $s < d$. The simulation of the noisy set is then performed by the convex sum of two states:

$$\rho_x \stackrel{!}{=} \alpha\rho_x^{(1)} + (1-\alpha)\frac{\mathbb{1}_{d-s}}{d-s} \tag{21}$$

The second state is always classically r-simulable for any $r \geq 1$. In order to simulate the first state, $\rho_x^{(1)}$, we follow a protocol analogous to the one given in SM II A but considering $s \leftrightarrow d$ and $r \leq s$:

1. We choose an arbitrary orthonormal basis of the space $\mathbb{C}^s$ spanned by the set.
2. We perform the same $s \times s$ unitary transformation U to each of the elements in the basis and take the one that overlaps the most with $|\psi_x\rangle$.
3. To simulate each $\rho_x^{(1)}$, we average over the Haar measure the as-above selected basis element.

Thus, we are claiming that

$$\rho_x^{(1)} = \int d\mu_{\text{Haar}}\left(U\right)\, |i_U^{(x)}\rangle\langle i_U^{(x)}| = \frac{H_r - 1}{s-1}\,|\psi_x\rangle\langle\psi_x| + \frac{s - H_r}{s-1}\frac{\mathbb{1}_s}{s}. \tag{22}$$

Hence, solving the system for (α, v) that arises from the matching

$$\alpha\rho_x^{(1)} + (1-\alpha)\frac{\mathbb{1}_{d-s}}{d-s} = v\,|\psi_x\rangle\langle\psi_x| + (1-v)\frac{\mathbb{1}_d}{d}, \tag{23}$$

leads to

$$\left.\begin{aligned} v &= \alpha\frac{H_r-1}{s-1} \\ \frac{1-v}{d} &= \frac{1-\alpha}{d-s} \end{aligned}\right\} \implies \begin{cases} \alpha = \dfrac{s-1}{d-1-H_r(d/s-1)} \\ v = \dfrac{H_r-1}{d-1-H_r(d/s-1)} = \dfrac{H_r-1}{d-1}\left(1+\dfrac{H_r(d-s)}{d(s-r)+s(H_r-1)}\right) \geq \dfrac{H_r-1}{d-1} \end{cases} \tag{24}$$

III. SUPPLEMENTARY NOTE 3: PROOF OF RESULT 2

We prove the optimality of the simulation described in II A when considering the set of all pure quantum states subject to isotropic noise,

$$\rho_\psi = v\,|\psi\rangle\!\langle\psi| + \frac{1-v}{d}\mathbb{1}_d. \tag{25}$$

We can parametrize this set by considering the computational basis $\{\,|a\rangle\}_{a=1}^{d}$ and any $d\times d$ unitary matrix:

$$\{\rho_U^{(a)}\}_{U\in\mathcal{U}(d)} = \left\{v\,U\,|a\rangle\!\langle a|\,U^\dagger + \frac{1-v}{d}\mathbb{1}_d\right\}_{U\in\mathcal{U}(d)}. \tag{26}$$

In addition, let us denote $F=\{\Phi_{i,\lambda},\,q_\lambda\}$ any set for which there exist a probability distribution $p(i|a,U,\lambda)$ fulfilling

$$\rho_U^{(a)} = \sum_{i,\lambda} p(i|a,U,\lambda)q_\lambda\Phi_{i,\lambda} \tag{27}$$

Under these conditions, a Lemma analogous to Lemma 1 in [2] can be stated:

Lemma 1. *Consider a group G with a unitary representation $\hat{U}(g)$ for each element $g\in G$ on the Hilbert space in hand. Say that for each ψ*

$$\rho_{U(g)\psi} = U(g)\rho_\psi U(g)^\dagger. \tag{28}$$

Then there exists a G-covariant optimal set: $\forall g\in G$, $F^\star=\{\Phi_\xi^\star,\,q_\xi^\star\}=\{U(g)\Phi_\xi^\star U(g)^\dagger,\,q_\xi^\star\}$.

The proof follows the same steps as the one for Lemma 1 in [2], so we omit it and refer to [2] for further details. In the case in hand, the symmetry group coincides with the unitary group in dimension d and the only unitary invariant optimal set to be considered is $F^\star=\{\,|\psi\rangle\!\langle\psi|\,,\,d\mu_{\text{Haar}}(\psi)\}$. Hence,

$$\rho_U^{(a)} \overset{!}{=} \int d\mu_{\text{Haar}}(\psi)p(\psi|a,U)\,|\psi\rangle\!\langle\psi| \tag{29}$$

subject to the constraints

$$\begin{aligned}\sum_{a=1}^{d}\rho_U^{(a)} = \mathbb{1}_d &\implies \sum_{a=1}^{d} p(\psi|a,U) = d,\\ \mathrm{Tr}\left\{\rho_U^{(a)}\right\} = 1 &\implies \int d\mu_{\text{Haar}}(\psi)p(\psi|a,U) = 1.\end{aligned} \tag{30}$$

In addition, since

$$\langle a|U^\dagger\rho_U^{(a)}U|a\rangle = \frac{(d-1)v+1}{d} \tag{31}$$

is a monotonically increasing function of v, in order to get the maximal value of v it is only needed to maximize the function

$$\int d\mu_{\text{Haar}}(\psi)p(\psi|a,U)|\,\langle a|\psi\rangle|^2. \tag{32}$$

First we notice that without loss of generality, we can choose $p(\psi|a,U)$ to be proportional to a deterministic probability distribution over a: $p(\psi|a,U)=d\,\mathcal{D}(a|U,\psi)$, whose support in the space of quantum states we denote by S_a. Due to normalization conditions (30), we must have

$$\int d\mu_{\text{Haar}}(\psi)\mathcal{D}(a|U,\psi) = \frac{1}{d} \implies \mu_{\text{Haar}}\,(S_a) = \int_{S_a} d\mu_{\text{Haar}}(\psi) = \frac{1}{d}. \tag{33}$$

Therefore, integral (32) reads

$$d\int d\mu_{\text{Haar}}(\psi)\mathcal{D}(a|U,\psi)|\,\langle a|\psi\rangle|^2 = d\int_{S_a} d\mu_{\text{Haar}}(\psi)|\,\langle a|\psi\rangle|^2 \tag{34}$$

Besides, by definition of deterministic strategy:

$$\forall a' \neq a \quad S_a \cap S_{a'} = \varnothing \quad \text{and} \quad \bigcup_a S_a = \mathcal{H}. \tag{35}$$

Moreover, let us introduce the sets

$$\chi_a = \left\{ |\psi\rangle : \quad |\langle a|\psi\rangle|^2 > |\langle a'|\psi\rangle|^2 \quad \forall\, |a'\rangle \perp |a\rangle \right\}. \tag{36}$$

Furthermore, it is clear that up to a set of null Haar measure, given any $|\psi\rangle$, $\exists!\, a$ such that $|\psi\rangle \in \chi_a$. Hence, integral (34) becomes

$$\sum_{\tilde{a}} \int_{S_a \cap \chi_{\tilde{a}}} d\mu_{\text{Haar}}(\psi)\, |\langle a|\psi\rangle|^2. \tag{37}$$

Let us now introduce in each addend the unitary transformation $P_{\tilde{a}}$ that permutes the states a and $\tilde{a}$, leaving the rest invariant:

$$\sum_{\tilde{a}} \int_{S_a \cap \chi_{\tilde{a}}} d\mu_{\text{Haar}}(\psi)\, |\langle a|P_{\tilde{a}} P_{\tilde{a}}|\psi\rangle|^2. \tag{38}$$

Applying the properties of the Haar measure, these integrals are equivalent to those in which the integrand and the domain of integration change accordingly:

$$\sum_{\tilde{a}} \int_{S_{\tilde{a}} \cap \chi_a} d\mu_{\text{Haar}}(\psi)\, |\langle \tilde{a}|\psi\rangle|^2. \tag{39}$$

Since the domain of integration of each term is now a subset of χ_a, we know that $|\langle \tilde{a}|\psi\rangle|^2 < |\langle a|\psi\rangle|^2$. Therefore each integral verifies

$$\int_{S_{\tilde{a}} \cap \chi_a} d\mu_{\text{Haar}}(\psi)\, |\langle \tilde{a}|\psi\rangle|^2 \leq \int_{S_{\tilde{a}} \cap \chi_a} d\mu_{\text{Haar}}(\psi)\, |\langle a|\psi\rangle|^2 \tag{40}$$

and the equality only holds for the term $\tilde{a} = a$. Up to this point, we have bounded from above the integral (32) by the sum

$$d \sum_{\tilde{a}} \int_{S_{\tilde{a}} \cap \chi_a} d\mu_{\text{Haar}}(\psi)\, |\langle a|\psi\rangle|^2. \tag{41}$$

We further notice that due to $S_a \cap S_{a'} = \varnothing\ \forall a \neq a'$, the sets $S_{\tilde{a}} \cap \chi_a$ in the sum are pairwise disjoint. Thus, since the integrand in each term is the same, we can join every integral in the sum to get

$$d \int_{(\bigcup_{\tilde{a}} S_{\tilde{a}}) \cap \chi_a} d\mu_{\text{Haar}}(\psi)\, |\langle a|\psi\rangle|^2. \tag{42}$$

The domain of integration is then $(\bigcup_{\tilde{a}} S_{\tilde{a}}) \cap \chi_a = \mathcal{H} \cap \chi_a = \chi_a$ up to a set of null Haar measure. In consequence,

$$d \int_{(\bigcup_{\tilde{a}} S_{\tilde{a}}) \cap \chi_a} d\mu_{\text{Haar}}(\psi)\, |\langle a|\psi\rangle|^2 = d \int_{\chi_a} d\mu_{\text{Haar}}(\psi)\, |\langle a|\psi\rangle|^2, \tag{43}$$

which corresponds to the integral associated with the optimal probability distribution $p^\star(\psi|a,U) = d\,\mathcal{D}^\star(a|U,\psi)$ with $S_a = \chi_a$ and

$$\mathcal{D}^\star(a|U,\psi) = \begin{cases} 1 & \text{if } |\langle a|\psi\rangle|^2 > |\langle a'|\psi\rangle|^2 \quad \forall\, |a'\rangle \perp |a\rangle \\ 0 & \text{otherwise.} \end{cases} \tag{44}$$

Summarizing, we have proven that

$$\int d\mu_{\text{Haar}}(\psi) p(\psi|a,U)\, |\langle a|\psi\rangle|^2 \leq \int d\mu_{\text{Haar}}(\psi) p^\star(\psi|a,U)\, |\langle a|\psi\rangle|^2. \tag{45}$$

So the maximal value of v is attained for the set $F^\star$ and the probability distribution $p^\star(\psi|a,U)$, which coincides with the strategy shown in II A (after identifying $a \leftrightarrow i$):

$$\int d\mu_{\text{Haar}}(U) \left| \langle \psi_x | i_U^{(x)} \rangle \right|^2 = d \int_1 d\mu_{\text{Haar}}(U)\, |\langle \psi_x|U|1\rangle|^2 = d \int_1 d\mu_{\text{Haar}}(\psi)\, |\langle \psi|1\rangle|^2. \tag{46}$$

In the proof for this appendix, we have considered $r = d$, but an analogous proof holds for $r \leq d$.

IV. SUPPLEMENTARY NOTE 4: PROOF OF RESULT 3

Let $\mathcal{E} = \left\{ \{\rho_i^{(j)}\}_{i=0}^{d-1} \right\}_{j=1}^{M}$ be the set of the $d\,M$ states obtained by mixing with white noise M bases of a d-dimensional Hilbert space, $\mathcal{A}_j = \left\{ |e_i^{(j)}\rangle \right\}_{i=0}^{d-1}$ for $j = 1, \ldots, M$, i.e.

$$\rho_i^{(j)} = v\,|e_i^{(j)}\rangle\langle e_i^{(j)}| + \frac{1-v}{d}\mathbb{1}, \tag{47}$$

for some visibility $v \in [0,1]$. In this section, we present a protocol for a classical simulation of complexity r of this set using the bases $\{\mathcal{A}_j\}_{j=1}^{M}$.

Consider that each simulation device randomly picks one of the M bases and randomly selects r elements of that basis. Defined $\mathcal{B}_\mu = \{\,|e_{\mu_1}\rangle, \ldots, |e_{\mu_r}\rangle\}$, with $\mu = 1, \ldots, n_{\text{sub}}$, with $n_{\text{sub}} = \binom{d}{r}$, the possible selections of r elements, we assume that each simulation device $\Pi_{j,\mu}$ can emit one of the following states:

$$\Pi_{j,\mu} \quad \longrightarrow \quad \begin{cases} |e_{\mu_1}^{(j)}\rangle\langle e_{\mu_1}^{(j)}|, & x = 1, \\ \quad\vdots & \\ |e_{\mu_r}^{(j)}\rangle\langle e_{\mu_r}^{(j)}|, & x = r, \\ & \\ \dfrac{1}{r}\displaystyle\sum_{l\in\mathcal{B}_\mu} |e_l^{(j)}\rangle\langle e_l^{(j)}|, & x = r+1, \\ \quad\vdots & \\ \dfrac{1}{r}\displaystyle\sum_{l\in\mathcal{B}_\mu} |e_l^{(j)}\rangle\langle e_l^{(j)}|, & x = d\,M. \end{cases} \tag{48}$$

Consider that we want to simulate the state $\rho_i^{(j)}$. If the associated pure state $|e_i^{(j)}\rangle$ belongs to the basis that was selected and is contained in the set $\mathcal{B}_\mu$, then $\Pi_{j,\mu}$ outputs exactly the state $|e_i^{(j)}\rangle$. If this is not the case, the simulation device randomly outputs one of the selected r states, i.e. $1/r\sum_{l\in\mathcal{B}_\mu} |e_l^{(j)}\rangle\langle e_l^{(j)}|$.

Label with $s = 1, \ldots, n_{\text{incl}}$ the subspaces of the basis j that include $|e_i^{(j)}\rangle$, where $n_{\text{incl}} = \binom{d-1}{r-1}$. Then, by selecting each box with a uniform probability distribution $q(j,\mu) = \frac{1}{M}\frac{1}{n_{\text{sub}}}$, the state $\rho_i^{(j)}$ will be simulated as

$$\rho_i^{(j)} = \frac{1}{M\,n_{\text{sub}}}\left[\sum_{s=1}^{n_{\text{incl}}} |e_i^{(j)}\rangle\langle e_i^{(j)}| + \frac{1}{r}\sum_{k=1}^{n_{\text{sub}}-n_{\text{incl}}}\sum_{l\in\mathcal{B}_k} |e_l^{(j)}\rangle\langle e_l^{(j)}| + \frac{1}{r}\sum_{\substack{y=1\\ y\neq j}}^{M}\sum_{\mu=1}^{n_{\text{sub}}}\sum_{l\in\mathcal{B}_\mu} |e_l^{(y)}\rangle\langle e_l^{(y)}|\right], \tag{49}$$

where $k = 1, \ldots, (n_{\text{sub}} - n_{\text{incl}})$ label the subsets $\mathcal{B}_k = \left\{ |e_{k_1}^{(j)}\rangle, \ldots, |e_{k_r}^{(j)}\rangle \right\}$ not including $|e_i^{(j)}\rangle$. Recalling the Pascal's rule, we find

$$\binom{d}{r} = \binom{d-1}{r-1} + \binom{d-1}{r} \quad \Longrightarrow \quad n_{\text{sub}} - n_{\text{incl}} = \binom{d-1}{r}. \tag{50}$$

Therefore, in the second term of (49), the summation over $k = 1, \ldots, (n_{\text{sub}} - n_{\text{incl}})$ and $l \in \mathcal{B}_k$ produces $\binom{d-2}{r-1}$ times each $|e_{l\neq i}^{(j)}\rangle\langle e_{l\neq i}^{(j)}|$. The first two terms of (49) become

$$\sum_{s=1}^{n_{\text{incl}}} |e_i^{(j)}\rangle\langle e_i^{(j)}| + \frac{1}{r}\sum_{k=1}^{n_{\text{sub}}-n_{\text{incl}}}\sum_{l\in\mathcal{B}_k} |e_l^{(j)}\rangle\langle e_l^{(j)}| = \left[n_{\text{incl}} - \frac{1}{r}\binom{d-2}{r-1}\right] |e_i^{(j)}\rangle\langle e_i^{(j)}| + \frac{1}{r}\binom{d-2}{r-1}\mathbb{1}, \tag{51}$$

where we added and subtracted $\frac{1}{r}\binom{d-2}{r-1}|e_i^{(j)}\rangle\langle e_i^{(j)}|$ to recover the identity. By inserting (51) in (49) and comparing it with the expression of the depolarised state (47), we directly get the visibility:

$$v = \frac{1}{M\,n_{\text{sub}}}\left[n_{\text{incl}} - \frac{1}{r}\binom{d-2}{r-1}\right] = \frac{1}{M}\,\frac{r-1}{d-1}. \tag{52}$$

To complete the protocol, we need to check that the remaining terms in (49) give the white noise contribution in (47), i.e.

$$\frac{1-v}{d}\mathbb{1} = \frac{1}{M\,n_{\text{sub}}}\frac{1}{r}\left[\binom{d-2}{r-1}\mathbb{1} + \sum_{\substack{y=1\\y\neq j}}^{M}\sum_{\mu=1}^{n_{\text{sub}}}\sum_{l\in\mathcal{B}_\mu}|e_l^{(y)}\rangle\langle e_l^{(y)}|\right]. \tag{53}$$

In the second term on the r.h.s. of (53), the summation over $\mu = 1,\ldots,n_{\text{sub}}$ and $l \in \mathcal{B}_\mu$ gives $\binom{d-1}{r-1}$ times each $|e_l^{(j)}\rangle\langle e_l^{(j)}|$. Then, summing over the bases $y \neq j$, we get

$$\frac{1}{M\,n_{\text{sub}}}\frac{1}{r}\left[\binom{d-2}{r-1}\mathbb{1} + (M-1)\binom{d-1}{r-1}\right]\mathbb{1} = \frac{M(d-1)-r+1}{M(d-1)}\frac{\mathbb{1}}{d}, \tag{54}$$

that is exactly $\frac{1-v}{d}\mathbb{1}$ with the visibility derived in (52).

The same result can also be derived in a more intuitive way. Again we consider that each simulation device randomly picks one of the M bases, randomly selects r elements of that basis and emits exactly the state $|e_i^{(j)}\rangle$ if it is included in the r elements selection; otherwise it randomly outputs one of the other states.

By averaging over all the simulation devices, this leads to the following simulation:

$$\rho_i^{(j)} = \frac{1}{M}\frac{r}{d}|e_i^{(j)}\rangle\langle e_i^{(j)}| + \frac{1}{M}\frac{d-r}{d}\left(\frac{1}{d-1}\sum_{k\neq i}|e_k^{(j)}\rangle\langle e_k^{(j)}|\right) + \left(1-\frac{1}{M}\right)\frac{1}{d}\mathbb{1}. \tag{55}$$

Here the first term corresponds to the case in which the state $|e_i^{(j)}\rangle$ is produced: on average, this happens with probability $\frac{1}{M}\frac{r}{d}$, since with probability $\frac{1}{M}$ we choose the correct basis, and with probability $\frac{r}{d}$ the basis element $|e_i^{(j)}\rangle$ is part of the r elements that were randomly selected. The second term arises when the correct basis is selected but the element $|e_i^{(j)}\rangle$ is not among the r selected ones, which happens with probability $\frac{1}{M}\frac{d-r}{d}$. In this case, each simulation device randomly produces one of the r selected states: therefore, on average, the state produced is $\frac{1}{d-1}\sum_{k\neq i}|e_k^{(j)}\rangle\langle e_k^{(j)}|$. Lastly, if the wrong basis is selected (that happens with probability $1-\frac{1}{M}$), the simulation device just produces a random state, that on average gives the maximally mixed state in dimension d.

Now, by using that for a fixed orthonormal basis j we have $\sum_{k\neq i}|e_k^{(j)}\rangle\langle e_k^{(j)}| = \mathbb{1} - |e_i^{(j)}\rangle\langle e_i^{(j)}|$, we can compute:

$$\begin{aligned}
\rho_i^{(j)} &= \frac{1}{M}\frac{r}{d}|e_i^{(j)}\rangle\langle e_i^{(j)}| + \frac{1}{M}\frac{d-r}{d}\left(\frac{1}{d-1}\sum_{k\neq i}|e_k^{(j)}\rangle\langle e_k^{(j)}|\right) + \left(1-\frac{1}{M}\right)\frac{1}{d}\mathbb{1}\\
&= \frac{1}{M}\frac{r}{d}|e_i^{(j)}\rangle\langle e_i^{(j)}| + \frac{1}{M}\frac{d-r}{d}\frac{1}{d-1}\left(\mathbb{1} - |e_i^{(j)}\rangle\langle e_i^{(j)}|\right) + \left(1-\frac{1}{M}\right)\frac{1}{d}\mathbb{1}\\
&= \frac{1}{M}\left(\frac{r}{d} - \frac{d-r}{d(d-1)}\right)|e_i^{(j)}\rangle\langle e_i^{(j)}| + \left(1-\frac{1}{M}+\frac{1}{M}\frac{d-r}{d-1}\right)\mathbb{1}/d\\
&= \frac{1}{M}\frac{r-1}{d-1}|e_i^{(j)}\rangle\langle e_i^{(j)}| + \left(1-\frac{1}{M}\frac{r-1}{d-1}\right)\mathbb{1}/d,
\end{aligned} \tag{56}$$

that is exactly the state in (47) with the visibility derived in (52).

V. SUPPLEMENTARY NOTE 5: NUMERICAL SEARCH FOR CLASSICAL MODELS

In this Appendix, we explain how to extend the numerical method for classical simulation presented in the main text in Section II E and we analyze different approaches for the selection of the unitaries. In particular, we will present how the method can also take into account the additional degree of complexity given by the r-dimensional restriction of the Hilbert space.

Let $\mathbf{b}_U = \{U\,|i\rangle\}_{i=1}^d$ be some basis of $\mathbb{C}^d$, defined by the unitary U, and construct the r-dimensional subspaces considering all possible selections $\mathbf{t}_U^{(\mu)} = \{U\,|k^{(\mu)}\rangle\}_{k=1}^r$ of r vectors from the basis, with $\mu = 1, \dots, \binom{d}{r}$. In this way, each preparation device $\mathcal{P}_\lambda$ is now identified by the tuple (U, μ). Since we want the r-dimensional states emitted by each $\mathcal{P}_{(U,\mu)}$ to commute, we can impose that they are diagonal in the same basis, i.e. $\tau_{x,(U,\mu)} = \sum_{k=1}^r p(k|x,U,\mu)\,U\,|k^{(\mu)}\rangle$. Then, given a set of states subject to white noise, the problem of finding a classical simulation can be reformulated in terms of the following linear program (LP):

$$\begin{aligned}
\max_{v,q,\tilde{p}} \quad & v \\
\text{s.t.} \quad & v\rho_x + \frac{1-v}{d}\mathbb{1} = \sum_{U\in\mathcal{U}} \sum_{\mu=1}^{\binom{d}{r}} \sum_{k=1}^{r} \tilde{p}(k|x,U,\mu)\,U\,|k^{(\mu)}\rangle\,, \quad \forall x, \\
& \tilde{p}(k|x,U,\mu) \geq 0 \quad \forall k, x, U, \mu, \\
& \sum_{k=1}^{r} \tilde{p}(k|x,U,\mu) = q(U,\mu), \quad q(U,\mu) \geq 0, \quad \forall x, U, \mu, \\
& \sum_{U\in\mathcal{U}} \sum_{\mu=1}^{\binom{d}{r}} q(U,\mu) = 1.
\end{aligned} \tag{57}$$

We emphasise that this method only uses isotropic noise as a quantifier of the simulability, i.e. only for $v = 1$ do we have a simulation of the original set. Importantly, that original set $\{\rho_x\}$ can correspond to arbitrary mixed states, i.e. they are not limited to isotropic noise. Although is generally applicable, the result of the simulation highly relies on the choice of the set of unitaries $U \in \mathcal{U}$. Moreover, it has access to a limited number of preparation devices, while, in principle, the optimal simulation could use an arbitray and possibly uncountable number of them. Therefore, choosing good unitaries is of crucial importance. Below we propose three different approaches for selecting the unitaries used in the classical simulation. All of them can lead to simulation models that outperform the analytical models given in the main text.

A. Random unitaries

The simplest way to evaluate the LP in (57) consists in generating multiple sets of random unitaries, $\{(U \in \mathcal{U})^{(j)}\}_{j=1}^n$, where n is the number of samples, and solve the optimization for each of these sets. This process can be repeated many times and the best result selected. Our implementation is available at [3]. It uses YALMIP [4] and the package QETLAB [5].

B. Optimization over unitaries

Although the random sampling method of SM V A may give good results, it does not try to select the unitaries in any systematic way. Here we propose a possible approach to address this task by also optimising over the choice of unitaries.

In this case, having fixed the number of unitaries to be used, instead of randomly choosing them to solve the linear program,

we also consider them as variables of an optimization problem, i.e.

$$
\begin{aligned}
\max_{U} \quad \max_{v,q,\tilde{p}} \quad & v \\
\text{s.t.} \quad & v\rho + \frac{1-v}{d^n}\mathbb{1} = \sum_{U}\sum_{\mu=1}^{\binom{d}{r}}\sum_{k=1}^{r} \tilde{p}(k|x,U,\mu)\, U\, |k^{(\mu)}\rangle\,, \quad \forall x, \\
& \tilde{p}(k|x,U,\mu) \geq 0 \quad \forall k,x,U,\mu, \\
& \sum_{k=1}^{r} \tilde{p}(k|x,U,\mu) = q(U,\mu), \quad q(U,\mu) \geq 0, \quad \forall x,U,\mu, \\
& \sum_{U}\sum_{\mu=1}^{\binom{d}{r}} q(U,\mu) = 1,
\end{aligned} \tag{58}
$$

By using the function `UC.m` [6], we parametrize each unitary in dimension d with a vector of d^2 elements, i.e.

$$
U = \begin{bmatrix} u_{11} & u_{12} & u_{13} & \dots & u_{1d} \\ u_{21} & u_{22} & u_{23} & \dots & u_{2d} \\ \vdots & \vdots & \vdots & \ddots & \vdots \\ u_{d1} & u_{d2} & u_{d3} & \dots & u_{dd} \end{bmatrix} \quad \leftrightarrow \quad x = \begin{bmatrix} u_{11} \\ u_{12} \\ \vdots \\ u_{1d} \\ u_{21} \\ \vdots \\ u_{2d} \\ \vdots \\ u_{dd} \end{bmatrix}. \tag{59}
$$

This allows us to rewrite the problem in (58) in a form that can be solved by the `fmincon` function in MATLAB [7]. Our implementation is available at [3].

C. Optimization over unitaries with additional constraints

The optimization over unitaries presented in SM V B can be modified imposing specific symmetries for the simulation devices. In this case, we start with a given set of unitaries and then optimize over their unitary transformations. This procedure turns out to be particularly useful when the set has a specific symmetric structure.

As an example, let us consider the $m = d$ set comprised of $m-1$ computational basis states, i.e. $\{\,|k\rangle\}_{k=0}^{d-2}$, and the uniform superposition state, i.e. $|e_0\rangle = \frac{1}{\sqrt{d}}\sum_{k=0}^{d-1}|k\rangle$. Given the high-symmetric structure of this set, the optimization over the unitaries is not always able to beat the analytical bounds with a reasonable number of preparation devices. However, starting from high-symmetric simulation devices and then optimizing over their unitary transformations can improve the results. For example, in this case we can choose the simulation devices to be the MUBs [8] in dimension d, i.e. $\{U_i \in \text{MUB}(d)\}_{i=1}^{d+1}$ and solve the optimization problem

$$
\begin{aligned}
\max_{A\in\mathcal{U}(d)} \quad \max_{v,q,\tilde{p}} \quad & v \\
\text{s.t.} \quad & v\rho + \frac{1-v}{d^n}\mathbb{1} = \sum_{\{AU_iA^\dagger\}}\sum_{\mu=1}^{\binom{d}{r}}\sum_{k=1}^{r} \tilde{p}(k|x,AU_iA^\dagger,\mu)\, AU_iA^\dagger\, |k^{(\mu)}\rangle\,, \quad \forall x, \\
& \tilde{p}(k|x,AU_iA^\dagger,\mu) \geq 0 \quad \forall k,x,AU_iA^\dagger,\mu, \\
& \sum_{k=1}^{r} \tilde{p}(k|x,AU_iA^\dagger,\mu) = q(AU_iA^\dagger,\mu), \quad q(AU_iA^\dagger,\mu) \geq 0, \quad \forall x,AU_iA^\dagger,\mu, \\
& \sum_{AU_iA^\dagger}\sum_{\mu=1}^{\binom{d}{r}} q(AU_iA^\dagger,\mu) = 1,
\end{aligned} \tag{60}
$$

where A is a generic unitary of the group $\mathcal{U}(d)$. Our implementation is available at [3].

D. Performance comparison

We can estimate the performance of the three approaches presented above by looking at some examples.

Set	$v(\mathrm{RU}_4)$	$v(\mathrm{UO}_4)$	$v(\mathrm{MUB}_4)$	$v(\mathrm{RU}_{20})$	$v(\mathrm{UO}_{20})$	Result 1
$\mathcal{E}_1$	0.2221	0.5142	0.6138	0.7131	0.8270	0.4167
$\mathcal{E}_2$	0.0984	0.1974	0.4367	0.4795	0.5821	0.3208
$\mathcal{E}_3$	0.0272	0.0885	0.5000	0.2675	0.3410	0.3208

$$\begin{aligned} &\mathcal{E}_1 = \{\rho(x)\}_{x=1}^{5}, \quad \rho(x) \in \mathbb{C}^3, \text{ available at [3]} \\ &\mathcal{E}_2 = \{\rho(x)\}_{x=1}^{3}, \quad \rho(x) \in \mathbb{C}^5, \text{ available at [3]} \\ &\mathcal{E}_3 = \{\,|k\rangle\langle k|\}_{k=1}^{4} \cup \{\,|e_0\rangle\langle e_0|\}, \quad |k\rangle \in \mathbb{C}^5,\ |e_0\rangle = \frac{1}{\sqrt{5}}\sum_{k=1}^{5}|k\rangle\,. \end{aligned} \tag{61}$$

Supplementary Table I: Performance comparison of the three approaches for numerical classical simulation of the quantum sets in (61). Here v is the critical visibility, and RU, UO and MUB denote the approaches presented in sections V A, V B and V C respectively. For the RU approach, $n = 50$ samplings have been used. Result 1 denotes the analytical bound found using Equation (9) in the main text. The subindex in e.g. $v(\mathrm{RU}_4)$ denotes the number of simulation devices used to perform the classical simulation.

$\mathcal{E}_1$ and $\mathcal{E}_2$ are random sets obtained using the `RandomDensityMatrix.m` function [3]. From Table I we can notice that, when the number of simulation devices is small (in particular, smaller or equal than the number of MUBs [8] in that dimension), the optimization method presented in V C outperforms the ones presented in V B and V A. This happens because for a small number of simulation devices, a structured set of unitaries (like the ones built from MUBs or SIC-POVMs [9]) covers the entire Hilbert space better than a set of non-structured unitaries. The situation changes when the number of simulation devices increases: for both $\mathcal{E}_1$ and $\mathcal{E}_2$, randomizing or optimizing over 20 unitaries outperforms the results given by the MUBs.

This is no longer true when the set itself has a specific structure. The results related to $\mathcal{E}_3$ show that even with 20 unitaries, the unitary randomization (V A) and the unitary optimization (V B) methods are far from reaching the results obtained by the MUB structured optimization method in V C.

VI. SUPPLEMENTARY NOTE 6: CONNECTIONS TO QUANTUM STEERING

A. Proof of Result 5

Consider a witness testing the classicality of a quantum set of qubit states. Following section II F in the main text, a witness is characterised by a set of real coefficients $\{c_{bxy}\}$ and a set of measurements $\{M_{b|y}\}$. We now select these measurements to be standard basis measurements, i.e. $M_{b|y}$ are rank-one and projective. Since the measurements have binary outcomes, we select the coefficients be of the form $c_{bxy} = (-1)^b s_{x,y}$ for some real coefficients $s_{x,y}$. Hence our witness function reads

$$W(\mathcal{E}) = \sum_{x,y} \sum_{b=0,1} (-1)^b s_{x,y} \operatorname{tr}\big(\rho_x M_{b|y}\big). \tag{62}$$

We can now use Result 4 to express the largest value of W achievable when the set admits a classical model.

$$\max_{\mathcal{E}\in\mathcal{S}} W(\mathcal{E}) = \max_{\gamma} \max_{\{\phi_0,\phi_1\}} \sum_{abxy} D_\gamma(a|x) \langle \phi_a | (-1)^b s_{x,y} M_{b|y} | \phi_a \rangle = \max_{\gamma} \max_{\{\Phi_0,\Phi_1\}} \sum_{abxy} D_\gamma(a|x) (-1)^b s_{x,y} \operatorname{tr}\big(\Phi_a M_{b|y}\big), \tag{63}$$

with $\Phi_a = |\phi_a\rangle\langle\phi_a|$. We use $\Phi_0 + \Phi_1 = \mathbb{1}$ to write this as

$$\max_{\mathcal{E}\in\mathcal{S}} W(\mathcal{E}) = \max_{\gamma} \max_{\{\Phi_0\}} \left[\sum_{abxy} D_\gamma(a|x)(-1)^{a+b} s_{x,y} \operatorname{tr}\big(\Phi_0 M_{b|y}\big) + \sum_{abxy} D_\gamma(a|x)(-1)^b s_{x,y} \operatorname{tr}\big(M_{b|y}\big) \right]. \tag{64}$$

The second term vanishes because $\operatorname{tr}\big(M_{0|y}\big) = \operatorname{tr}\big(M_{1|y}\big) = 1$ due to rank-one projectivity. The maximisation over Φ_0 can then be expressed as

$$\max_{\mathcal{E}\in\mathcal{S}} W(\mathcal{E}) = \max_{\gamma} \max_{\{\Phi_0\}} \sum_{abxy} D_\gamma(a|x)(-1)^{a+b} s_{x,y} \operatorname{tr}\big(\Phi_0 M_{b|y}\big) = \max_{\gamma} \lambda_{\max} \left(\sum_{abxy} D_\gamma(a|x)(-1)^{a+b} s_{x,y} M_{b|y} \right), \tag{65}$$

where λ_{max} denotes the largest eigenvalue.

Let us now derive the expression for the bound ζ in the full-correlation steering inequality

$$\tilde{W} = \sum_{xy} s_{x,y} \langle A_x, B_y \rangle_\rho \leq \zeta, \tag{66}$$

where we will select Bob's measurements as identical to those used in the set witness, i.e. $B_{b|y} \equiv M_{b|y}$. Alice's measurements have binary outcomes. Expanding the right-hand side and defining $\sigma_{a|x} = \operatorname{tr}_A(\rho(A_{a|x} \otimes \mathbb{1}))$,

$$\tilde{W} = \sum_{abxy} (-1)^{a+b} s_{x,y} \operatorname{tr}\big(\rho A_{a|x} \otimes M_{b|y}\big) = \sum_{abxy} (-1)^{a+b} s_{x,y} \operatorname{tr}\big(\sigma_{a|x} M_{b|y}\big). \tag{67}$$

If the assemblage $\{\sigma_{a|x}\}$ is non-steerable, then it admits a local hidden state model $\sigma_{a|x} = \sum_\gamma p(a|x,\gamma) q_\gamma \sigma_\gamma$. Thus,

$$\zeta = \max \sum_{\gamma} q_\gamma \sum_{abxy} p(a|x,\gamma)(-1)^{a+b} s_{x,y} \operatorname{tr}\big(\sigma_\gamma M_{b|y}\big) = \max_{\gamma,\sigma_\gamma} \sum_{abxy} D_\gamma(a|x)(-1)^{a+b} s_{x,y} \operatorname{tr}\big(\sigma_\gamma M_{b|y}\big), \tag{68}$$

where in the second step we have used that the optimal value is achieved for a determinsitic input-output strategy for Alice. For each γ, the optimal value corresponds to a max-eigenvalue calculation,

$$\zeta = \max_{\gamma} \lambda_{\max} \left(\sum_{abxy} D_\gamma(a|x)(-1)^{a+b} s_{x,y} M_{b|y} \right). \tag{69}$$

This expression is identical to that in Equation (65) obtained for the set classicality witness.

VII. SUPPLEMENTARY NOTE 7: PROOF OF RESULT 6

A. Classical dimensionality implies joint measurability for binarizations

We show a connection between any classically simulable set $\mathcal{E} = \{\rho_x\}_x \subset \mathcal{L}\left(\mathbb{C}^d\right)$ and joint measurability. Let us first define the extended set $\mathcal{E}' = \mathcal{E} \cup \{\frac{\mathbb{1}-\rho_x}{d-1}\}_x$. We show that $\mathcal{E}$ is classically simulable if and only if $\mathcal{E}'$ is classically simulable. The necessary condition is trivial, since any simulation for $\mathcal{E}'$ is as well a simulation for $\mathcal{E}$. Regarding the sufficient condition, $\mathcal{E}$ is classically simulable if by definition

$$\rho_x = \int d\lambda\, q\left(\lambda\right) \tau_{x,\lambda} \quad = \int d\lambda\, q\left(\lambda\right) \sum_{i=1}^{d} p\left(i|x\,\lambda\right)\, |\phi_{i\,\lambda}\rangle\langle\phi_{i\,\lambda}|\,, \tag{70}$$

where $\{\phi_{i\,\lambda}\}_i$ are orthonormal basis states given by preparation device $\mathcal{P}_\lambda$. We build the simulation for $\{\frac{\mathbb{1}-\rho_x}{d-1}\}_x$ by

$$\frac{\mathbb{1}-\rho_x}{d-1} = \int d\lambda\, q\left(\lambda\right) \sum_{i=1}^{d} \frac{1-p\left(i|x\,\lambda\right)}{d-1}\, |\phi_{i\,\lambda}\rangle\langle\phi_{i\,\lambda}|\,. \tag{71}$$

We notice that for each λ we have used the same basis $\{\phi_{i\,\lambda}\}_i$ as in Equation (70) and that $\{\frac{1-p(i|x\,\lambda)}{d-1}\}_i$ constitutes a proper probability distribution since it is non-negative and adds up to the identity. Hence, we have built a classical simulation for $\mathcal{E}'$.

Secondly, we show that if $\mathcal{E}$ is classically simulable, then the measurements $\mathcal{M} = \{M_{0|x} = \rho_x, M_{1|x} = \mathbb{1} - \rho_x\}_x$ are jointly measurable. Since $\mathcal{E}$ is classically simulable, so is $\mathcal{E}'$ with an explicit simulation given by Eqs. (70) and (71). Therefore, we define the parent measurement for $\mathcal{M}$ by $G(i,\lambda) = q\left(\lambda\right)\, |\phi_{i\,\lambda}\rangle\langle\phi_{i\,\lambda}|$ and we consider the probability distribution $p_{i\,\lambda}\left(0|x\right) = p\left(i|x\,\lambda\right)$, $p_{i\,\lambda}\left(1|x\right) = 1 - p\left(i|x\,\lambda\right)$. Thus, it is clear that

$$\begin{aligned} M_{0|x} &= \int d\lambda\, \sum_{i=1}^{d} p_{i\,\lambda}\left(0|x\right) G(i,\lambda), \\ M_{1|x} &= \int d\lambda\, \sum_{i=1}^{d} p_{i\,\lambda}\left(1|x\right) G(i,\lambda), \end{aligned} \tag{72}$$

with

$$\begin{aligned} &\int d\lambda \sum_{i=1}^{d} G(i,\lambda) = \int d\lambda\, q\left(\lambda\right) \sum_{i=1}^{d} |\phi_{i\,\lambda}\rangle\langle\phi_{i\,\lambda}| = \mathbb{1}, \\ &p_{i\,\lambda}\left(0|x\right) + p_{i\,\lambda}\left(1|x\right) = 1, \end{aligned} \tag{73}$$

which concludes the proof for joint measurability.

B. Equivalence for qubits

Let us see that for qubits the relation is an equivalence. Given the set $\mathcal{E} = \{\rho_x\}_x$, we consider the associated measurements $\mathcal{M} = \{M_{0|x} = \rho_x, M_{1|x} = \mathbb{1} - \rho_x\}_x$. If $\mathcal{M}$ is jointly measurable,

$$\begin{aligned} \rho_x = M_{0|x} &= \sum_{\lambda} p_\lambda\left(0|x\right) G\left(\lambda\right), \\ \mathbb{1} - \rho_x = M_{1|x} &= \sum_{\lambda} p_\lambda\left(1|x\right) G\left(\lambda\right). \end{aligned} \tag{74}$$

In addition, since $G(\lambda)$ is a general positive semi-definite qubit operator fulfilling $\sum_\lambda G(\lambda) = \mathbb{1}$, it can be parametrized by

$$\begin{aligned} &G(\lambda) = p\left(\lambda\right) \mathbb{1} + p\left(\lambda\right) \eta_\lambda\, \vec{n}_\lambda \cdot \vec{\sigma}, \quad \sum_\lambda p\left(\lambda\right) = 1, \\ &0 \leq \eta_\lambda \leq 1, \quad \|\vec{n}_\lambda\|^2 = 1, \quad \sum_\lambda p\left(\lambda\right) \eta_\lambda\, \vec{n}_\lambda = 0. \end{aligned} \tag{75}$$

Therefore,

$$\begin{aligned} \rho_x &= \sum_\lambda p_\lambda(0|x)\, p(\lambda)\, \mathbb{1} + \sum_\lambda p_\lambda(0|x)\, p(\lambda)\, \eta_\lambda\, \vec{n}_\lambda \cdot \vec{\sigma}, \\ \mathbb{1} - \rho_x &= \sum_\lambda p_\lambda(1|x)\, p(\lambda)\, \mathbb{1} + \sum_\lambda p_\lambda(1|x)\, p(\lambda)\, \eta_\lambda\, \vec{n}_\lambda \cdot \vec{\sigma}. \end{aligned} \tag{76}$$

with $\sum_\lambda p_\lambda(0|x)\, p(\lambda) = \sum_\lambda p_\lambda(1|x)\, p(\lambda) = 1/2$ by normalization of the ρ_x states. Moreover, since $p_\lambda(0|x) + p_\lambda(1|x) = 1$, we must have that either $p_\lambda(0|x) \leq 1/2$ or $p_\lambda(1|x) \leq 1/2$. Without loss of generality we assume $p_\lambda(0|x) \leq 1/2$ and we focus on giving a classical simulation for $\mathcal{E}$.

The states $\{\rho_x\}_x$ are classically simulable if

$$\rho_x = \sum_\nu q(\nu) \sum_{i=1}^{2} q(i|x\,\nu)\; |\phi_{i\,\nu}\rangle\langle\phi_{i\,\nu}|\,. \tag{77}$$

In terms of Bloch vectors, we have

$$\rho_x = \frac{1}{2}\mathbb{1} + \frac{1}{2}\sum_\nu q(\nu)\, \mu_{x\,\nu}\, \vec{u}_\nu \cdot \vec{\sigma}, \tag{78}$$

where $\vec{u}_\nu$ is the Bloch vector of $|\phi_{1\,\nu}\rangle$, which coincides with the opposite of that of $|\phi_{2\,\nu}\rangle$ (since $|\phi_{1\,\nu}\rangle$ and $|\phi_{2\,\nu}\rangle$ are orthogonal to each other). The coefficient $\mu_{x\,\nu}$ is defined by $\mu_{x\,\nu} = 2q(1|x\,\nu) - 1$. Equating this last expression for ρ_x with that derived from joint measurability in Equation (76), yields to

$$\begin{aligned} &\sum_\lambda p_\lambda(0|x)\, p(\lambda) = 1/2, \\ &\sum_\lambda p(\lambda)\, p_\lambda(0|x)\, \eta_\lambda\, \vec{n}_\lambda = \frac{1}{2}\sum_\nu q(\nu)\, \mu_{x\,\nu}\, \vec{u}_\nu. \end{aligned} \tag{79}$$

The first condition is trivially satisfied, while for the second to hold we make the choice $\nu = \lambda$ and

$$q(\lambda) = p(\lambda)\,, \quad \mu_{x\,\lambda} = 2p_\lambda(0|x)\, \eta_\lambda, \quad \vec{u}_\lambda = \vec{n}_\lambda, \tag{80}$$

which is well defined since $2p_\lambda(0|x) \leq 1$. Hence, under this assignment, a simulation for $\mathcal{E}$ is given.

If on the contrary we had had $p_\lambda(1|x) \leq 1/2$, we would have proceeded as before but simulating $\{\mathbb{1} - \rho_x\}_x$, which for $d = 2$ is a properly normalized state. The consequent simulation for $\mathcal{E}$ is derived from the classical simulation of the extended set $\mathcal{E}'$.

[1] H. M. Wiseman, S. J. Jones, and A. C. Doherty, Steering, entanglement, nonlocality, and the einstein-podolsky-rosen paradox, Phys. Rev. Lett. **98**, 140402 (2007).

[2] S. J. Jones, H. M. Wiseman, and A. C. Doherty, Entanglement, einstein-podolsky-rosen correlations, bell nonlocality, and steering, Phys. Rev. A **76**, 052116 (2007).

[3] Code for classical simulation of quantum ensembles 10.5281/zenodo.17474952 (2024).

[4] J. Löfberg, Yalmip : A toolbox for modeling and optimization in matlab, in *In Proceedings of the CACSD Conference* (Taipei, Taiwan, 2004).

[5] N. Johnston, QETLAB: A MATLAB toolbox for quantum entanglement, version 0.9, `https://qetlab.com` (2016).

[6] C. Spengler, Composite parameterization of unitary groups (2011).

[7] Find minimum of constrained nonlinear multivariable function.

[8] T. Durt, B.-G. Englert, I. Bengtsson, and K. Życzkowski, On mutually unbiased bases, International Journal of Quantum Information **08**, 535–640 (2010).

[9] J. M. Renes, R. Blume-Kohout, A. J. Scott, and C. M. Caves, Symmetric informationally complete quantum measurements, Journal of Mathematical Physics **45**, 2171–2180 (2004).
